# Plasmid-mediated *mcr-1* in carbapenem-susceptible *Escherichia coli* ST156 causing a blood infection: an unnoticeable spread of colistin resistance in Brazil?

**DOI:** 10.6061/clinics/2017(10)09

**Published:** 2017-10

**Authors:** Flavia Rossi, Raquel Girardello, Carlos Morais, Ana Paula Cury, Layla Farage Martins, Aline Maria da Silva, Edson Abdala, João Carlos Setubal, Alberto José da Silva Duarte

**Affiliations:** I Divisao Laboratorio Central – LIM03, Setor de Microbiologia, Hospital das Clinicas HCFMUSP, Faculdade de Medicina, Universidade de Sao Paulo, Sao Paulo, SP, BR; II Departamento de Bioquímica, Instituto de Química, Universidade de Sao Paulo, Sao Paulo, SP, BR; III Nucleo de Apoio a Pesquisa em Biologia Computacional e Genomica (NUBIC), Universidade de Sao Paulo, Sao Paulo, SP, BR; IV Instituto do Cancer do Estado de Sao Paulo, Hospital das Clinicas HCFMUSP, Faculdade de Medicina, Universidade de Sao Paulo, Sao Paulo, SP, BR

**Keywords:** Colistin Resistance, Polymyxin Resistance, *Escherichia Coli*, Carbapenem-Susceptible, ST156

## Abstract

**OBJECTIVE:**

We describe an IncX4 pHC891/16mcr plasmid carrying *mcr-1* in a colistin-resistant and carbapenem-susceptible *E. coli* isolate (HC891/16), ST156, which caused a blood infection in a Brazilian patient with gallbladder adenocarcinoma.

**METHODS:**

Strain HC891/16 was subjected to whole genome sequencing using the MiSeq Platform (Illumina, Inc., USA). Assembly was performed using Mira and ABACAS.

**RESULTS:**

The isolates showed resistance only to ciprofloxacin, ampicillin and cefoxitin, and whole-genome sequencing revealed the presence of *aac(6′)Ib-cr* and *bla*_TEM1_.

**CONCLUSION:**

Our findings warn of the possible silent dissemination of colistin resistance by carbapenem-susceptible *mcr-1* producers, as colistin susceptibility is commonly tested only among carbapenem-resistant isolates.

Liu et al. 1 reported the emergence of an *mcr-1* plasmidial resistance mechanism to colistin in *E. coli* from China, which was followed by many reports worldwide 2-7. In Brazil, Fernandes et al. 8 reported an IncX4 plasmid carrying *mcr-1* in *E. coli* isolated from a soft tissue infection in a human. These authors also described evidence that *E. coli* harboring *mcr-1* has been occurring in food-producing animals in Brazil since at least 2012 9. However, to date, there is no consensus in the scientific community regarding the clinical importance of this new colistin resistance mechanism, as it confers a low level of colistin resistance compared with chromosomal mechanisms and appears to have been present for a long time 10. Epidemiological data can be used to elucidate the impact of this plasmidial mechanism for the dissemination of colistin resistance. In this study, we describe a plasmid-mediated *mcr-1* in a colistin-resistant and carbapenem-susceptible *E. coli* isolate that caused a blood infection in a cancer patient from Brazil.

ECF, a 75-year-old female with a gallbladder adenocarcinoma diagnosed in January 2016, was admitted to Hospital das Clínicas, São Paulo for investigation, and a hepatic drain was placed. Bile cultures were positive for *Enterococcus faecalis* susceptible to all drugs. One month later, she developed fever and presented to the emergency room (ER). Blood cultures were positive for *E. coli* phenotypically classified as an ESβL-producer that was susceptible to carbapenem and aminoglycosides, resistant to ciprofloxacin (MIC>4 mg/L) and with a colistin MIC≤0.5 mg/L. She received meropenem with good clinical evolution. One month later, she began ambulatory chemotherapy (cisplatin and gemcitabine). On April 11, 2016, she returned to the ER with a high fever, leukocytosis, and elevated CRP, and her bloodcultures were positive for *E. coli* with a different phenotypic profile. It was susceptible to amikacin (MIC, ≤2 mg/L), gentamicin (MIC, 2 mg/mL), cefuroxime (MIC, 4 mg/L), ceftazidime (MIC, ≤1 mg/L), ceftriaxone (MIC, ≤1 mg/L), cefepime (MIC, ≤1 mg/L), ertapenem (MIC, ≤0.5 mg/L), imipenem (MIC, ≤0.25 mg/L), meropenem (MIC, ≤0.25 mg/L), piperacillin/tazobactam (MIC, ≤4 mg/L) and tigecycline (MIC, ≤0.5 mg/L); intermediate to cefoxitin (MIC, 16 mg/L); and resistant to ampicillin (MIC, >32 mg/L) and ciprofloxacin (MIC, >4 mg/L). The colistin MIC was 4 mg/L. This strain was positive for *mcr-1* by PCR using specific primers as previously described 1 and was denominated HC891/16. The patient received antibiotics and support medication and was discharged with no infection. The identification and antimicrobial susceptibility tests of the isolates were performed by Vitek-MS and Vitek-2 (bioMeriéux, France), respectively, and colistin susceptibility was confirmed by CLSI broth microdilution 11. Strain HC891/16 was subjected to whole genome sequencing using the MiSeq Platform (Illumina, Inc., USA). The sequencing library was prepared from 40 ng of HC891/16 strain total DNA using the Illumina Nextera DNA library preparation kit (Illumina, Inc., USA). After quantification of the resulting library using the KAPA Library Quantification Kit, a sequencing run was performed in the Illumina MiSeq to generate 250 bp paired-end reads. Assembly was performed using Mira 12 and ABACAS 13, resulting in 71 contigs encompassing the main chromosome (5,053,377 bp) and at least one plasmid (33,227 bp). Genome sequences were deposited in GenBank under accession number MRDN01000000. The presence of resistance genes was determined with ResFinder 14.

The strain HC891/16 belongs to sequence type (ST) 156. *E. coli* ST156 harboring the *mcr-1* gene was previously detected in one duck sample, from China, showing a carbapenem-resistant profile 15. The *mcr-1* gene is in pHC891/16mcr, a 33.2-kb plasmid, which is classified as incompatibility group IncX4. This plasmid shows 100% nucleotide identity to pICBEC7Pmcr (CP017246.1), and it is found in animal samples from Latin America. Furthermore, 99% nucleotide identity was observed when this plasmid was compared with the following previously described conjugative plasmids harboring the *mcr-1* gene: pOW3E1 (KX129783.1), detected in environmental and animal samples; pMCR1.2-IT (KX236309.1), harboring a *mcr-1.2* variant of *K. pneumoniae* from a human infection in Italy; pmcr1_IncX4 (KU761327.1), found in clinical isolates from China; pMCR1-NJ-IncX4 (KX447768.1), found in a carbapenem-resistant *E. coli* isolate harboring *mcr-1* and *bla*_NDM-5_, causing a complicated urinary tract infection in the USA 16-19. The IncX4 plasmid appears to be globally disseminated among environmental, food and human *mcr-1* producer strains, including KPC-2 and a *mcr*-1 producer-*K. pneumoniae* strain that have been recently reported in Brazil 20. In addition to *mcr-1*, we also detected the presence of genes *aac(6′)Ib-cr* and *bla*_TEM1_ in the HC891/16 strain.

In a previous study from our institution, carbapenem susceptibility was observed in 21.5% of all colistin-resistant Gram-negative isolates examined in the last five years 21. It is important to highlight that the carbapenem-susceptible and *mcr-1-*positive phenotype may easily go unnoticed because many clinical laboratories in Brazil only test or release colistin susceptibility to clinicians when the isolates are carbapenem-resistant. Olaitan et al. 22 state that clinicians and microbiologists should be vigilant for the possible existence of colistin-resistant bacteria not only in patients undergoing colistin therapy but also in patients who are not receiving the drug, as resistant bacteria could later be selected if colistin is used. In Brazil, colistin has been used as a first-line drug in many critical care units due to high rates of carbapenem-resistance among the OXA-23-producer *Acinetobacter baumannii* and KPC- producer *K. pneumoniae* 23,24.

The dissemination of colistin resistance is a serious problem for infection control, as this antimicrobial is one of the last therapeutic options for infections caused by multidrug-resistant Gram-negative isolates. At this time, attention should be paid to this carbapenem-susceptible-*mcr-1* phenotype, as these isolates may be silently contributing to the spread of colistin resistance.

## AUTHOR CONTRIBUTIONS

Rossi F and Girardello R were responsible for the data analysis and preparation of the manuscript. Morais C, Martins LF, Silva AM, Setubal JC were responsible for the bioinformatics analysis. Cury AP was responsible for the data analysis. Abdala E was responsible for the description of clinical case. Duarte AJ was responsible for the preparation of the manuscript.
